# Distribution of angle alpha and angle kappa offsets among adult candidates for cataract surgery

**DOI:** 10.1007/s00417-024-06596-6

**Published:** 2024-08-06

**Authors:** Gil Neuman, Adi Abulafia, Lauren Wasser, David Zadok

**Affiliations:** Department of Ophthalmology, The Eisenberg R&D Authority, Shaare Zedek Medical Center, The Hebrew University of Jerusalem, Jerusalem, Israel

**Keywords:** Angle alpha, Angle kappa, Cataract, IOLMaster 700, Refractive surgery

## Abstract

**Purpose:**

The current study aimed to describe the distribution of angle alpha and angle kappa offsets as well as their associated ocular biometric parameters in a large population of candidates for cataract surgery.

**Methods:**

This cross-sectional retrospective study included 8,119 eyes of 4,781 candidates for cataract surgery (mean age 70.7 ± 12.9 years). There were 49.9% right eyes, and 53.0% patients were females. The angles offset and ocular biometric parameters were measured by the IOLMaster 700 (Carl Zeiss Meditec, AG, Germany).

**Results:**

Patient’s age and gender, and most of their ocular biometric measurements were similar for the right and left eyes except for pupil diameter (4.01 ± 1.18 vs. 3.92 ± 1.14 mm, respectively, *P *< 0.001). The angle alpha offset magnitude was similar for the right and left eyes (0.50 ± 0.20 and 0.51 ± 0.21 mm, *P *= 0.08), whereas the angle kappa offset magnitude was greater in the right eyes (0.37 ± 0.21 vs. 0.33 ± 0.20 mm, *P *< 0.001). The angle kappa offset magnitudes were greater in the right eyes compared to the left eyes for both males (0.36 ± 0.21 vs. 0.33 ± 0.21 mm, respectively, *P *< 0.001) and females (0.37 ± 0.20 vs. 0.34 ± 0.20 mm, respectively, *P *< 0.001). The offset magnitudes of both angles varied significantly according to gender, eye laterality, angle location, and biometric parameters (e.g., axial length). The offset magnitudes of both angles were positively correlated in both right and left eyes.

**Conclusions:**

The offset magnitudes of both the angle alpha and angle kappa present significant variations according to gender, eye laterality, angle location, and biometric parameters, such as AL. These values are also population-specific.

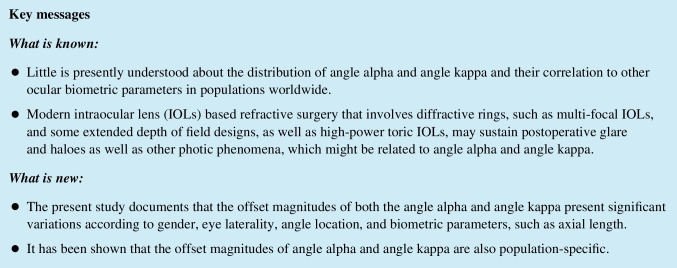



## Introduction

Advances in cataract and refractive surgery have raised patient expectations for complete refractive correction and optimal visual outcomes. Patients opting for modern intraocular lens (IOL)-based refractive surgery, including multifocal IOLs, extended depth of focus (EDOF) IOLs, and high-power toric IOLs, may experience postoperative glare, haloes, and other photic phenomena [1–3].

Angle alpha and angle kappa, representing misalignments between the eye’s axes, may be linked to these phenomena, potentially impacting visual quality [4–7]. Research suggests that a significant angle kappa correlates with increased glare and halo effects following multifocal IOL implantation [5]. Additionally, the magnitude of angle kappa offset has been associated with visual outcomes, including modulation transfer function cutoff, following trifocal diffractive IOL implantation [8]. In a study focusing on two aspheric IOLs, higher alpha angles were linked to elevated higher-order aberrations (HOAs) across all pupil sizes, and an increase in angle kappa was associated with decreased vision quality, as evidenced by a reduced Strehl ratio and increased internal HOAs [9]. Similarly, larger horizontal angle kappa and alpha were associated with greater horizontal decentration, while a deeper anterior chamber depth and larger vertical angle kappa predicted increased vertical decentration [10].

Accurate centration is crucial for successful laser refractive surgery. A decentered ablation can lead to postoperative glare, irregular astigmatic error, and reduced best spectacle-corrected visual acuity (BCVA). While pupil-centered laser ablation generally yields optimal refractive outcomes, it can pose challenges for individuals with high angle kappa values, potentially causing a significant discrepancy between the laser-ablated zone and the visual axis, resulting in less-than-optimal refractive results [11–14].

Currently, there is limited understanding regarding the global distribution of angle alpha and angle kappa and their relationship with other ocular biometric parameters. Swept-source optical coherence tomography (ssOCT) devices, such as the IOLMaster 700 (Carl Zeiss Meditec, AG, Germany), now offer measurement of these angles' offset magnitudes alongside other ocular biometric parameters [15]. This study aimed to characterize the distributions of angle alpha and angle kappa offset magnitudes, along with their associated ocular biometric parameters, in a large population of adult candidates for cataract surgery.

## Materials and methods

This cross-sectional retrospective study adhered to the tenets of the Declaration of Helsinki and received institutional review board approval. Patient consent was waived due to the retrospective de-identified nature of the study.

### Patients

Included in this study were individuals referred for cataract surgery between February 2018 and January 2020, who were 18 years of age and older, and who had undergone ocular biometric evaluation by means of the IOLMaster 700 (Carl Zeiss Meditec, AG, Germany, software version: 1.80.6.60340.C75999). Criteria for eligibility included no machine-defined failed data points for biometric parameters, with the exception of blank values in axis-related biometric parameters or repeat keratometry measurements. Non-phakic eyes and those that had undergone refractive surgery or vitrectomy were excluded. For eyes with more than one eligible measurement, the measurement with minimum blank values for axis-related biometric parameters or repeat keratometry measurements was chosen. Both right and left eyes were included in this study, however all of the statistical analyses were performed separately for each group.

### Assessment

The clinical assessment included an evaluation of corrected visual acuity, a standard preoperative evaluation, and ocular biometry measurement with the IOLMaster 700. The ocular biometric parameters included lens thickness (LT), pupil diameter (PD), mean corneal power (Kmean), white-to-white distance (WTW), anterior chamber depth (ACD), central corneal thickness (CCT), and axial length (AL). The IOLMaster 700 measurements were performed by a team of experienced technicians, under mesopic conditions. The right eye was measured before the left eye, approximately 30 seconds apart. Low-quality scans, as defined by the IOLMaster 700, were deleted. The IOLMaster 700 automatically defined the corneal center as well as the pupillary center, which were assumed to represent the optical axis and pupillary axis, respectively, as demonstrated by an anterior chamber capture. The visual axis was derived from the main corneal light reflex on that capture.

**Fig. 1:**
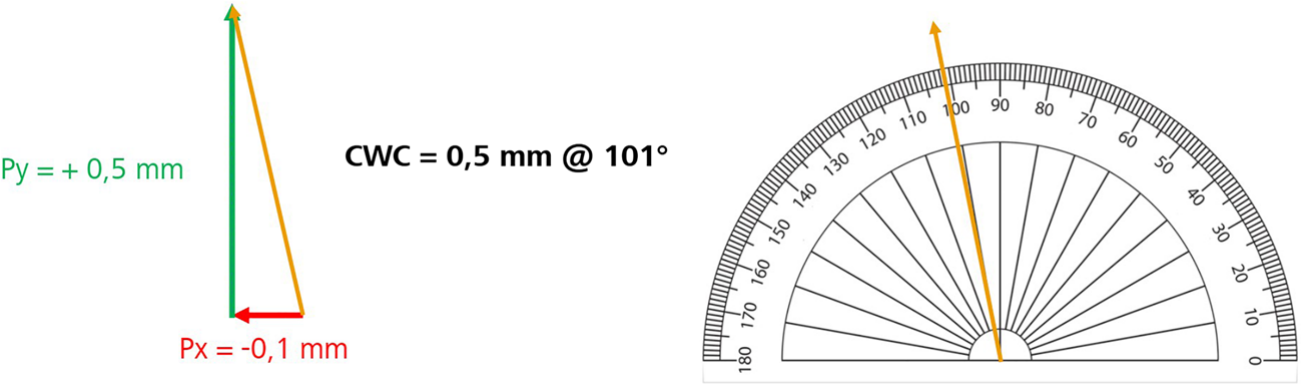
Illustration of the calculation of angle kappa offset magnitude. Carl Zeiss Meditec, AG, Germany. CWC, Chang-Waring Chord

The IOLMaster 700 output includes the values of Ix and Iy, which are the cartesian coordinates of the visual axis (corneal vertex) from the corneal center (“angle alpha offset”), and the cartesian coordinates of the visual axis (corneal vertex) from the pupil center (Px and Py; “angle kappa offset”) as well as the Chang-Waring Chord (CWC) distance and angle, which are the polar coordinates of the same angle offset (Fig. 1).

The visual axis was assumed by the IOLMaster 700 as the line that connects the fixation point, the front and rear nodal points, and the fovea. The offset magnitude of each angle was calculated as the square root of the sum of the squares of the cartesian horizontal and vertical components of the angle offset measurement. The orientation of angle alpha and kappa offsets was defined in relation to the corneal center or pupillary center, respectively, with positive values of the X and Y direction indicating a nasal and superior location of the angle offset in relation to the respective center. The angle offset location could then be divided into 4 quadrants (superior vs. inferior and nasal vs. temporal) according to the respective origins of each angle offset. In order to enable correct comparison between the right and left eyes, the values of Ix and Px of the left eyes were multiplied by -1 prior to analysis, since the nasal and temporal locations are mirrored between the right and left eyes, as opposed to the superior and inferior locations which remain on the same orientation.

Similar to Meng et al.’s study [16], the eyes included in the current study were further divided according to their AL (in mm) to groups 1-6, respectively: AL < 22, 22 ≤ AL < 24, AL 24 ≤ AL < 26, 26 ≤ AL < 28, 28 ≤ AL < 30 and AL ≥ 30.

The distributions of angle alpha and angle kappa offsets and their associations with various ocular parameters were assessed as well.

### Statistical analysis

Continuous variables were expressed as mean ± standard deviation. Categorical variables were expressed as frequencies (percentage). The data analyzed in our study were not normally distributed, therefore nonparametric tests were used to compare the continuous and categorical variables of 2 or multiple groups (Chi-square test of independence, Mann–Whitney *U* test, and Kruskal-Wallis test followed by a *post hoc* pairwise comparisons with the Wilcoxon rank-sum test). Spearman’s rank correlation was performed for correlation analysis. A multivariable logistic regression analysis was used to determine the odds ratio and significance of variables of interest in the angles’ offset magnitudes. In the multivariate logistic regression analysis, angles alpha of a offset magnitude < 0.45 mm and angles kappa of a offset magnitude < 0.30 mm was defined as 0 (small), whereas all other angles were defined as 1 (large). All tests were 2-tailed, with *P*-values < 0.05 being considered as significant. The analysis was performed with the R software (A Language and Environment for Statistical Computing, https://www.R-project.org) and Python 2.7 (Python Software Foundation, https://www.python.org). Graphics were created with R packages ggplot2 [17] and modelsummary [18], and Python package matplotlib [19].

## Results

A total of 8,119 eyes of 4,781 candidates for cataract surgery were included in this study, out of 18,171 records from the IOLMaster 700. The patients included 2,536 (53.0%) females, the mean age of the entire study was 70.7 ± 12.9 years (median 72.0 years; range 18–99 years; Table 1), and there were 4,051 (49.9%) right eyes.

**Table 1:** Demographic and Clinical Characteristics

	RE	LE	Overall	*P*-value
	(*N* = 4051)	(*N* = 4068)	(*N* = 8119)	
Age, y				
Mean ± SD	70.4 ± 12.8	71.0 ± 12.0	70.7 ± 12.4	0.14
Median [Min, Max]	72.0 [18.0, 99.0]	72.0 [18.0, 99.0]	72.0 [18.0, 99.0]	
Gender				
Female	2149 (53.0%)	2180 (53.6%)	4329 (53.3%)	0.641
Male	1902 (47.0%)	1888 (46.4%)	3790 (46.7%)	
AL, mm				
Mean ± SD	24.0 ± 1.72	23.9 ± 1.72	23.9 ± 1.72	0.422
Median [Min, Max]	23.7 [14.6, 35.9]	23.6 [15.0, 35.1]	23.6 [14.6, 35.9]	
AL group, mm				
AL < 22	238 (5.9%)	256 (6.3%)	494 (6.1%)	0.839
22 ≤ AL < 24	2223 (54.9%)	2236 (55.0%)	4459 (54.9%)	
24 ≤ AL < 26	1195 (29.5%)	1164 (28.6%)	2359 (29.1%)	
26 ≤ AL < 28	288 (7.1%)	298 (7.3%)	586 (7.2%)	
28 ≤ AL < 30	64 (1.6%)	74 (1.8%)	138 (1.7%)	
AL ≥ 30	43 (1.1%)	40 (1.0%)	83 (1.0%)	
Kmean, D				
Mean ± SD	44.1 ± 1.89	44.2 ± 1.89	44.2 ± 1.89	0.11
Median [Min, Max]	44.1 [33.2, 68.6]	44.1 [34.4, 67.4]	44.1 [33.2, 68.6]	
ACD, mm				
Mean ± SD	3.17 ± 0.436	3.16 ± 0.436	3.17 ± 0.436	0.106
Median [Min, Max]	3.16 [1.82, 5.55]	3.15 [1.78, 5.01]	3.16 [1.78, 5.55]	
LT, mm				
Mean ± SD	4.49 ± 0.485	4.51 ± 0.481	4.50 ± 0.483	0.091
Median [Min, Max]	4.50 [1.01, 6.55]	4.51 [1.13, 6.12]	4.51 [1.01, 6.55]	
CCT, mm				
Mean ± SD	0.538 ± 0.038	0.539 ± 0.038	0.539 ± 0.038	0.204
Median [Min, Max]	0.538 [0.267, 0.999]	0.539 [0.335, 1.06]	0.538 [0.267, 1.06]	
WTW, mm				
Mean ± SD	11.9 ± 0.452	11.9 ± 0.456	11.9 ± 0.454	0.292
Median [Min, Max]	11.9 [9.85, 15.0]	11.9 [9.80, 17.2]	11.9 [9.80, 17.2]	
PD, mm				
Mean ± SD	4.01 ± 1.18	3.92 ± 1.14	3.97 ± 1.16	**< 0.001**
Median [Min, Max]	3.84 [0.348, 10.6]	3.76 [0.383, 12.4]	3.80 [0.348, 12.4]	
Ix, mm				
Mean ± SD	0.435 ± 0.190	0.437 ± 0.198	0.436 ± 0.194	0.434
Median [Min, Max]	0.432 [-1.02, 1.81]	0.438 [-1.33, 1.80]	0.434 [-1.33, 1.81]	
Iy, mm				
Mean ± SD	0.045 ± 0.244	0.047 ± 0.256	0.046 ± 0.250	0.266
Median [Min, Max]	0.051 [-1.63, 1.71]	0.061 [-1.99, 1.93]	0.056 [-1.99, 1.93]	
Px, mm				
Mean ± SD	0.289 ± 0.222	0.229 ± 0.225	0.259 ± 0.226	**< 0.001**
Median [Min, Max]	0.285 [-1.18, 1.92]	0.227 [-1.42, 1.81]	0.256 [-1.42, 1.92]	
Py, mm				
Mean ± SD	0.020 ± 0.210	0.055 ± 0.216	0.038 ± 0.214	**< 0.001**
Median [Min, Max]	0.025 [-1.12, 1.64]	0.056 [-1.69, 2.14]	0.041 [-1.69, 2.14]	
Angle alpha offset, mm				
Mean ± SD	0.498 ± 0.198	0.506 ± 0.205	0.502 ± 0.201	0.081
Median [Min, Max]	0.478 [0.047, 1.87]	0.483 [0.034, 2.11]	0.480 [0.034, 2.11]	
Angle kappa offset, mm				
Mean ± SD	0.368 ± 0.205	0.334 ± 0.203	0.351 ± 0.204	**< 0.001**
Median [Min, Max]	0.343 [0.007, 1.93]	0.305 [0.005, 2.14]	0.325 [0.005, 2.14]	

RE, right eye; LE, left eye; AL, axial length; Kmean, mean corneal power; ACD, anterior chamber depth; LT, lens thickness; CCT, central corneal thickness; WTW, white-to-white distance; PD, pupil diameter; Ix and Iy, the cartesian coordinates of angle alpha offset; Px and Py, the cartesian coordinates of angle kappa offset

**Bold** indicates significant

### Baseline characteristics

**Fig. 2:**
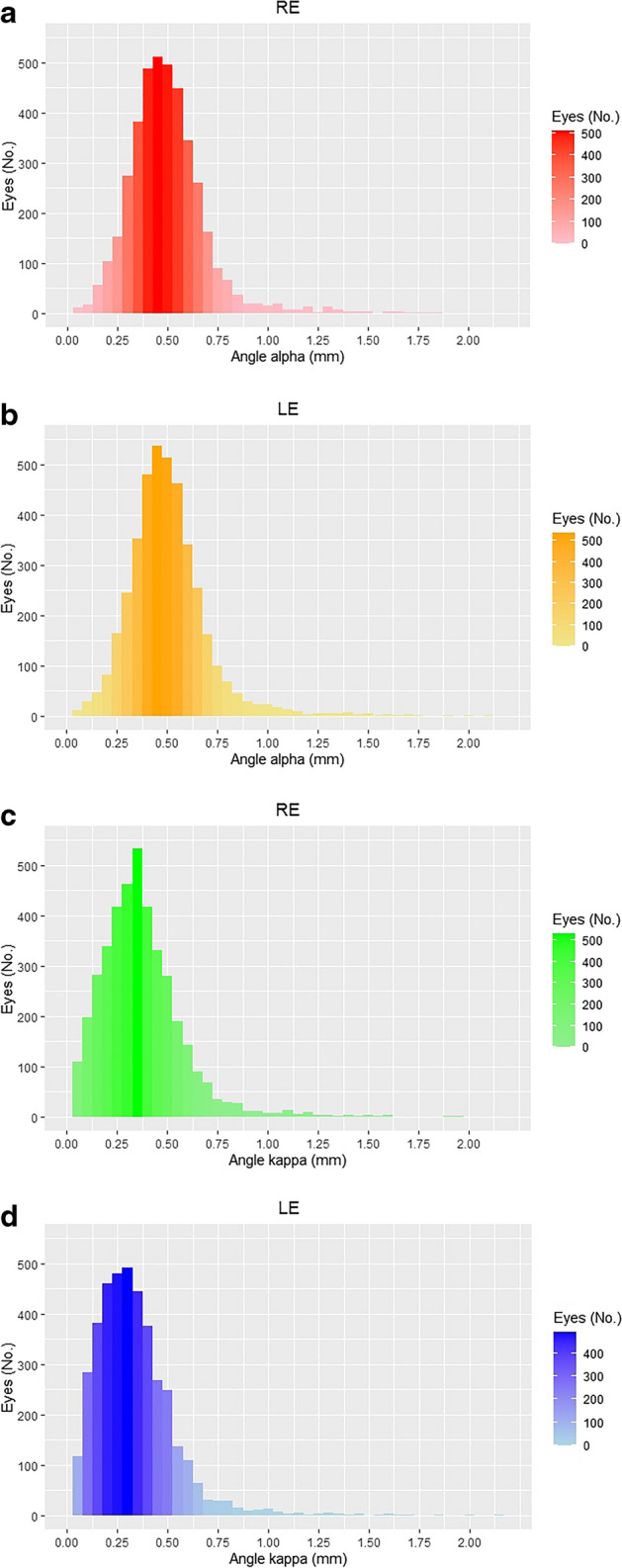
Frequency histogram of offset magnitudes of (**a**) angle alpha in right eyes, (**b**) angle alpha in left eyes, (**c**) angle kappa in right eyes, and (**d**) angle kappa in left eyes. RE, right eye; LE, left eye

Age, gender, and most of the ocular biometric measurements were similar between the right and left eyes (Table 1), but there was a significant difference in PD (4.01 ± 1.18 vs. 3.92 ± 1.14 mm, respectively, *P* < 0.001), which is clinically negligible in terms of pupil area. The offset magnitude of angle alpha was similar between the right and left eyes (0.50 ± 0.20 and 0.51 ± 0.21 mm, respectively, *P* = 0.08; Fig. 2). The offset magnitude of angle kappa was greater in the right eyes compared to the left eyes (0.37 ± 0.21 vs. 0.33 ± 0.20 mm, respectively, *P* < 0.001). A comparison of each coordinate of the offset angles separately (Ix and Iy of angle alpha and Px and Py of angle kappa) yielded similar results.

**Fig. 3:**
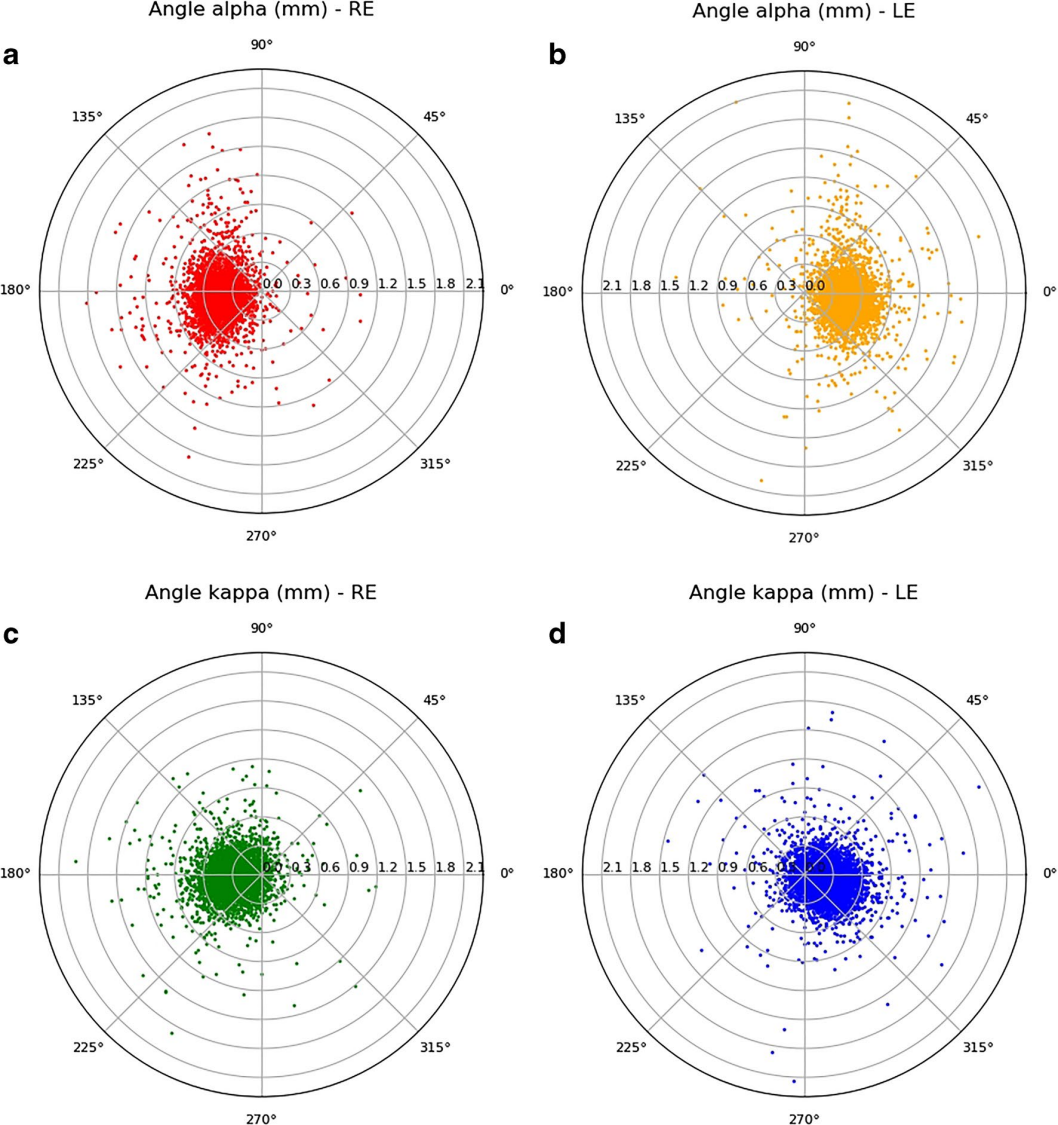
Polar scatter plot of offset magnitudes of (**a**) angle alpha in right eyes, (**b**) angle alpha in left eyes, (**c**) angle kappa in right eyes, and (**d**) angle kappa in left eyes. The origin represents the visual axis. RE, right eye; LE, left eye

Table 2 describes the distribution of angles alpha and kappa offsets according to quadrant orientation and axial length. Both angles’ offsets were located temporally to the visual axis (98.7% and 90.3% for angle alpha and angle kappa offsets; respectively) in most of the eyes (Fig. 3). More than 50% of the alpha and kappa angles’ offsets were located in the nasal-superior quadrant for both right and left eyes. The distributions of the angles’ offset’s location between the right and left eyes, however, were significantly different (*P* = 0.021 for angle alpha offset and *P* < 0.001 for angle kappa offset).

**Table 2:** Distribution of Angles’ Offsets According to Quadrant

	RE	LE	Overall	*P*-value*
	(*N* = 4051)	(*N *= 4068)	(*N* = 8119)	
Angle alpha offset quadrant				
Nasal-superior	2482 (61.3%)	2550 (62.7%)	5032 (62.0%)	**0.021**
Nasal-inferior	1522 (37.6%)	1456 (35.8%)	2978 (36.7%)	
Temporal-superior	31 (0.8%)	27 (0.7%)	58 (0.7%)	
Temporal-inferior	16 (0.4%)	35 (0.9%)	51 (0.6%)	
Angle kappa offset quadrant				
Nasal-superior	2142 (52.9%)	2279 (56.0%)	4421 (54.5%)	**< 0.001**
Nasal-inferior	1626 (40.1%)	1282 (31.5%)	2908 (35.8%)	
Temporal-superior	129 (3.2%)	282 (6.9%)	411 (5.1%)	
Temporal-inferior	154 (3.8%)	225 (5.5%)	379 (4.7%)	

RE, right eye; LE, left eye

*Statistical analysis by the Chi-square test of independence

**Bold** indicates significant

The distributions of the angle offset magnitude according to AL subgroups were similar for the right and left eyes (Table 2). The Kolmogorov–Smirnov test demonstrated that the distributions of the offset magnitudes for both alpha and kappa angles in this patient population were not normal but rather leptokurtic with positive skewness.

### Characteristics of both angles in the different locations and axial lengths

**Fig. 4:**
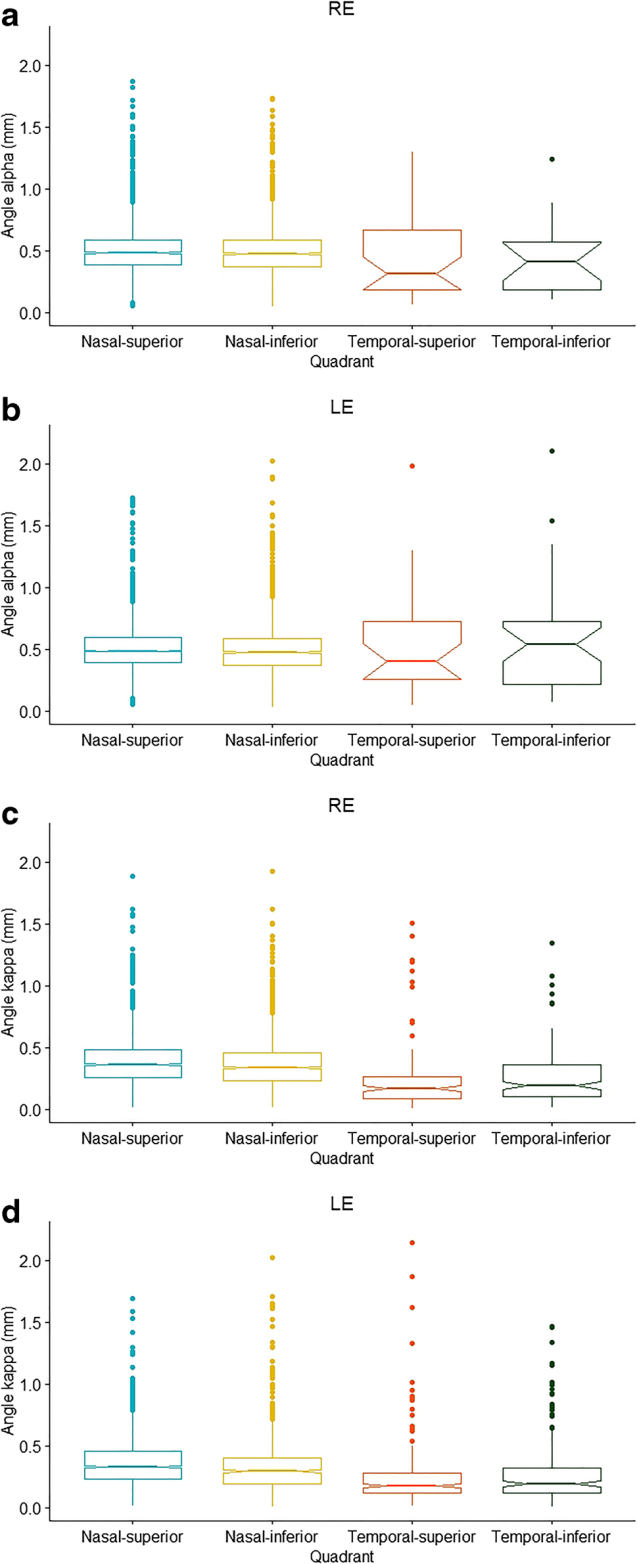
Comparison of angle offset magnitude according to quadrant location: (**a**) angle alpha in right eyes, (**b**) angle alpha in left eyes, (**c**) angle kappa in right eyes, and (**d**) angle kappa in left eyes. RE, right eye; LE, left eye

The offset magnitudes of angle alpha in the various quadrants were significantly different solely for the left eyes (*P* < 0.05; Table 3 and Fig. 4). The offset magnitudes of angle kappa were significantly greater when located nasally to the pupillary center compared to angles’ offset magnitudes located on the temporal side in both eyes individually (*P* < 0.001 for both). Further pairwise analysis revealed greater angle kappa offset magnitudes in the nasal-superior quadrant compared to the nasal-inferior quadrant. Angle alpha and angle kappa demonstrated different offset magnitudes in both eyes among the AL subgroups (*P* < 0.001 for all, Table 4). A gradual decrease in angle alpha offset magnitude was demonstrated in parallel with an increase in AL, starting from the largest values in group 1 (AL < 22 mm) and decreasing towards the nadir mean value in group 5 (28 ≤ AL < 30 mm; *P* < 0.001). This was followed by an increase in angle alpha offset magnitude in the eyes with an AL >30 mm. A similar trend of a gradual decrease was exhibited for angle kappa offset magnitude: the group 4 values (26 ≤ AL < 28 mm) were at the nadir, and there was a non-homogenous trend towards increase among the larger AL groups in the right and left eyes.

**Table 3:** Angle Alpha and Angle Kappa Offset Magnitudes in Different Quadrants

		Nasal-superior	Nasal-inferior	Temporal-superior	Temporal-inferior	Overall	*P*-value
RE	Angle alpha offset, mm	(*N* = 2482)	(*N* = 1522)	(*N* = 31)	(*N* = 16)	(*N* = 4051)	
	Mean ± SD	0.498 ± 0.187	0.499 ± 0.209	0.480 ± 0.373	0.461 ± 0.319	0.498 ± 0.198	0.142
	Median [Min, Max]	0.480 [0.051, 1.87]	0.472 [0.047, 1.73]	0.316 [0.063, 1.30]	0.409 [0.106, 1.24]	0.478 [0.047, 1.87]	
	Angle kappa offset, mm*	(*N* = 2142)	(*N* = 1626)	(*N* = 129)	(*N* = 154)	(*N* = 4051)	
	Mean ± SD	0.385 ± 0.197	0.367 ± 0.201	0.243 ± 0.268	0.258 ± 0.222	0.368 ± 0.205	**< 0.001**
	Median [Min, Max]	0.358 [0.011, 1.89]	0.340 [0.013, 1.93]	0.166 [0.007, 1.51]	0.189 [0.012, 1.35]	0.343 [0.007, 1.93]	
LE	Angle alpha offset, mm†	(*N* = 2550)	(*N* = 1456)	(*N* = 27)	(*N* = 35)	(*N* = 4068)	
	Mean ± SD	0.506 ± 0.183	0.502 ± 0.223	0.552 ± 0.436	0.589 ± 0.452	0.506 ± 0.205	**0.046**
	Median [Min, Max]	0.486 [0.054, 1.73]	0.474 [0.034, 2.02]	0.400 [0.05, 1.98]	0.538 [0.071, 2.11]	0.483 [0.034, 2.11]	
	Angle kappa offset, mm*	(*N* = 2279)	(*N* = 1282)	(*N* = 282)	(*N* = 225)	(*N* = 4068)	
	Mean ± SD	0.356 ± 0.180	0.326 ± 0.209	0.243 ± 0.247	0.278 ± 0.263	0.334 ± 0.203	**< 0.001**
	Median [Min, Max]	0.331 [0.015, 1.69]	0.293 [0.005, 2.02]	0.173 [0.011, 2.14]	0.191 [0.007, 1.47]	0.305 [0.005, 2.14]	

RE, right eye; LE, left eye

*P ≤ 0.001 difference between all groups, except between temporal-superior and temporal-inferior

†*P* ≤ 0.05 difference between nasal-superior and nasal-inferior

**Bold** indicates significant

**Table 4:** Angle Alpha and Angle Kappa Offset Magnitudes in Different Axial Length Groups

Axial length Group, mm		RE			LE		
		N	Angle alpha offset, mm*	Angle kappa offset, mm†	N	Angle alpha offset, mm‡	Angle kappa offset, mm§
1: AL < 22	Mean ± SD	238	0.564 ± 0.205	0.441 ± 0.220	256	0.585 ± 0.239	0.436 ± 0.260
	Median [Min, Max]		0.535 [0.151, 1.43]	0.408 [0.027, 1.62]		0.540 [0.071, 1.98]	0.387 [0.047, 2.14]
2: 22 ≤ AL < 24	Mean ± SD	2223	0.523 ± 0.186	0.390 ± 0.201	2236	0.533 ± 0.193	0.349 ± 0.195
	Median [Min, Max]		0.503 [0.051, 1.82]	0.364 [0.007, 1.93]		0.509 [0.054, 2.11]	0.323 [0.005, 2.02]
3: 24 ≤ AL < 26	Mean ± SD	1195	0.469 ± 0.200	0.334 ± 0.193	1164	0.470 ± 0.192	0.301 ± 0.184
	Median [Min, Max]		0.447 [0.047, 1.87]	0.312 [0.012, 1.56]		0.452 [0.052, 2.02]	0.274 [0.009, 1.71]
4: 26 ≤ AL < 28	Mean ± SD	288	0.399 ± 0.192	0.294 ± 0.194	298	0.413 ± 0.214	0.268 ± 0.215
	Median [Min, Max]		0.363 [0.063, 1.34]	0.272 [0.011, 1.25]		0.375 [0.05, 1.52]	0.213 [0.007, 1.47]
5: 28 ≤ AL < 30	Mean ± SD	64	0.373 ± 0.212	0.295 ± 0.216	74	0.374 ± 0.236	0.313 ± 0.197
	Median [Min, Max]		0.344 [0.071, 1.21]	0.237 [0.027, 1.12]		0.313 [0.034, 1.28]	0.306 [0.021, 1.02]
6: AL ≥ 30	Mean ± SD	43	0.460 ± 0.302	0.409 ± 0.321	40	0.422 ± 0.297	0.381 ± 0.288
	Median [Min, Max]		0.397 [0.072, 1.24]	0.311 [0.013, 1.35]		0.367 [0.082, 1.70]	0.292 [0.075, 1.42]
Overall	Mean ± SD	4051	0.498 ± 0.198	0.368 ± 0.205	4068	0.506 ± 0.205	0.334 ± 0.203
	Median [Min, Max]		0.478 [0.047, 1.87]	0.343 [0.007, 1.93]		0.483 [0.034, 2.11]	0.305 [0.005, 2.14]
*P*-value			**< 0.001**	**< 0.001**		**< 0.001**	**< 0.001**

RE, right eye; LE, left eye

*P ≤ 0.05 difference between each pair of Groups 1, 2, 3, 4, and 5, except between 4 to 5, and between Group 1, and 2 to Group 6

†*P* ≤ 0.05 difference between each pair of Groups 1, 2, 3, 4, and 5, except between 4 to 5

‡*P* ≤ 0.05 difference between each pair of Groups 1, 2, 3, 4, and 5, and between Groups 1, 2, and 3 to Group 6

§*P* ≤ 0.05 difference between each pair of Groups 1, 2, 3, and 4, and between Group 1, and 4 to Group 5, and between Group 1, and 4 to Group 6

**Bold** indicates significant

### Offset magnitude variation in concordance with gender and laterality

The offset magnitude of angle alpha in the male patients’ eyes was similar between the right and left eyes (0.48 ± 0.21 and 0.48 ± 0.21 mm, respectively,* P* = 0.675), while the right eyes of the female patients had a smaller angle alpha offset magnitude (0.51 ± 0.19 vs. 0.53 ± 0.20 mm, respectively, *P* < 0.05) (Table 5). In contrast, the angle kappa offset magnitudes were greater in the right eyes compared to the left eyes for both males (0.36 ± 0.21 vs. 0.33 ± 0.21 mm, respectively, *P* < 0.001) and females (0.37 ± 0.20 vs. 0.34 ± 0.20 mm, respectively, *P* < 0.001). Table 6 shows a sub-analysis with a comparison of the angle offset magnitudes according to eye side and gender. Angle alpha’s offset magnitude was greater in the female patient’s eyes than in the male eyes for both the right and left eyes (0.51 ± 0.19 vs. 0.48 ± 0.21 mm, respectively, *P* < 0.001 in right eyes, and 0.53 ± 0.20 vs. 0.48 ± 0.21 mm, respectively, *P* < 0.001 in the left eyes). The offset magnitude of angle kappa in the right and left eyes of the patients of both genders was not different.§

**Table 5:** Comparison of Angle Offset Magnitude According to Gender

	Male			Female		
	RE	LE	*P*-value	RE	LE	*P*-value
	(*N* = 1902)	(*N* = 1888)		(*N *= 2149)	(*N* = 2180)	
Angle alpha offset, mm						
Mean ± SD	0.482 ± 0.205	0.483 ± 0.208	0.675	0.511 ± 0.191	0.525 ± 0.200	**0.048**
Median [Min, Max]	0.455 [0.047, 1.87]	0.461 [0.034, 2.02]		0.492 [0.063, 1.82]	0.500 [0.054, 2.11]	
Angle kappa offset, mm						
Mean ± SD	0.363 ± 0.207	0.332 ± 0.205	**< 0.001**	0.372 ± 0.203	0.336 ± 0.201	**< 0.001**
Median [Min, Max]	0.337 [0.012, 1.89]	0.299 [0.007, 2.02]		0.348 [0.007, 1.93]	0.309 [0.005, 2.14]	

RE, right eye; LE, left eye

**Bold** indicates significant

**Table 6:** Comparison of Angle Offset Magnitude According to Laterality

	RE			LE		
	Female	Male	*P-*value	Female	Male	*P*-value
	(*N* = 2149)	(*N* = 1902)		(*N* = 2180)	(*N* = 1888)	
Angle alpha offset, mm						
Mean ± SD	0.511 ± 0.191	0.482 ± 0.205	**<0.001**	0.525 ± 0.200	0.483 ± 0.208	**<0.001**
Median [Min, Max]	0.492 [0.063, 1.82]	0.455 [0.047, 1.87]		0.500 [0.054, 2.11]	0.461 [0.034, 2.02]	
Angle kappa offset, mm						
Mean ± SD	0.372 ± 0.203	0.363 ± 0.207	0.064	0.336 ± 0.201	0.332 ± 0.205	0.23
Median [Min, Max]	0.348 [0.007, 1.93]	0.337 [0.012, 1.89]		0.309 [0.005, 2.14]	0.299 [0.007, 2.02]	

RE, right eye; LE, left eye

**Bold** indicates significant

### Parameters associated with angle alpha and angle kappa

**Fig. 5:**
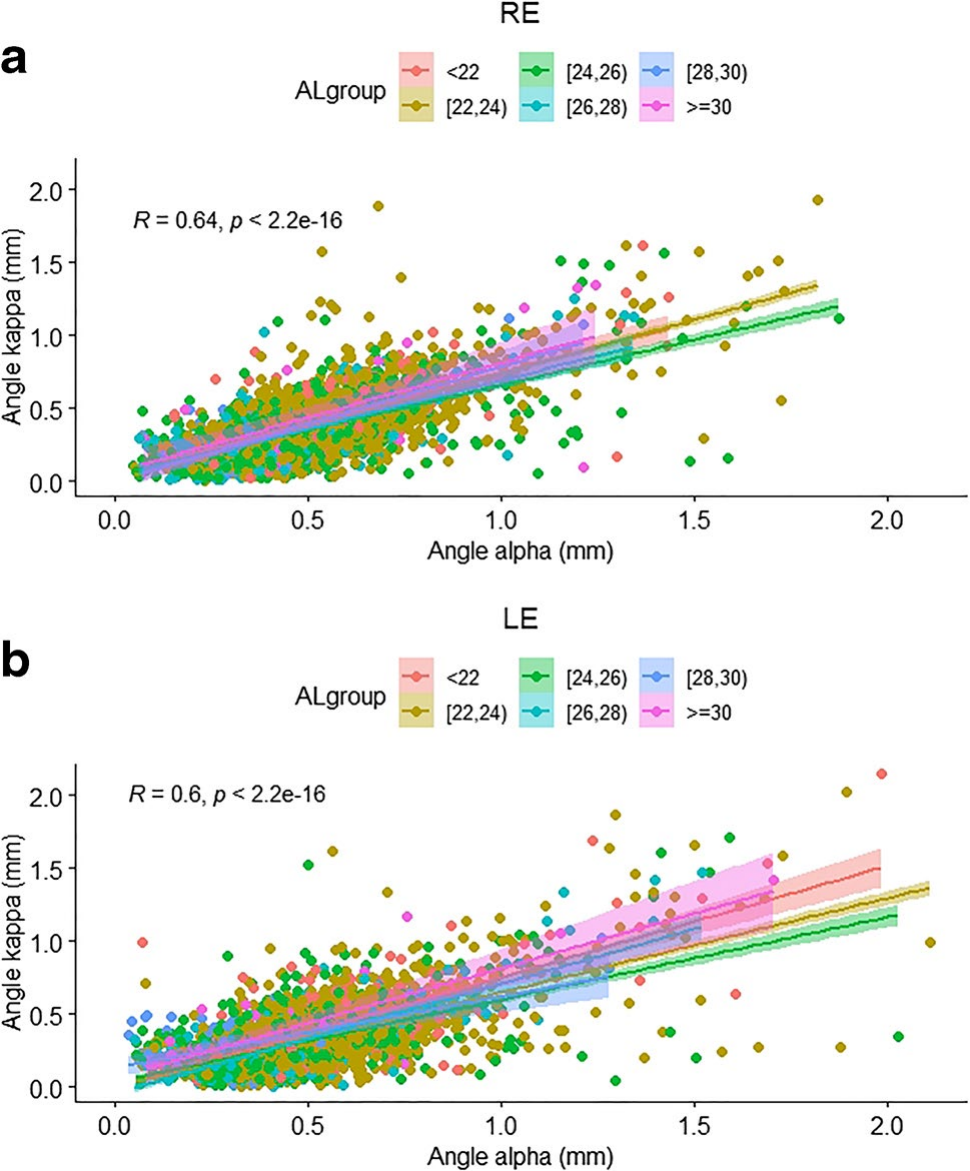
Angle alpha – angle kappa offset magnitudes correlation in (**a**) right eyes, and (**b**) left eyes, separated according to AL subgroups. RE, right eye; LE, left eye; AL, axial length

Table 7 demonstrates the Spearman's rank correlation coefficient of parameters associated with the offset magnitude of the angles in both the right and left eyes individually. The offset magnitude of angle alpha correlated positively with age and LT, and negatively with Kmean, WTW, ACD, and AL regardless of eye laterality (*P* < 0.001 for all). Similarly, the offset magnitude of angle kappa correlated positively with age, LT, and PD, and negatively with Kmean, WTW, ACD, and AL, regardless of eye laterality (*P* < 0.001 for all). These results are consistent with the positive correlation existing between the offset magnitudes of angle alpha and angle kappa in both the right and left eyes individually (r = 0.64 and r = 0.6, respectively, *P* < 0.001 for both) as shown in Fig. 5.

**Table 7:** Spearman's Rank Correlation Coefficient of Parameters Associated with Angle Offset Magnitude

	Angle alpha offset, mm				Angle kappa offset, mm			
	RE		LE		RE		LE	
	r	*P* value	r	*P* value	r	*P* value	r	*P* value
Age, y	0.079	**0.000**	0.093	**0.000**	0.102	**0.000**	0.130	**0.000**
LT, mm	0.094	**0.000**	0.072	**0.000**	0.145	**0.000**	0.158	**0.000**
PD, mm	0.012	0.457	-0.007	0.633	0.109	**0.000**	0.080	**0.000**
Kmean, D	-0.133	**0.000**	-0.127	**0.000**	-0.115	**0.000**	-0.109	**0.000**
WTW, mm	-0.151	**0.000**	-0.164	**0.000**	-0.079	**0.000**	-0.097	**0.000**
ACD, mm	-0.274	**0.000**	-0.255	**0.000**	-0.291	**0.000**	-0.288	**0.000**
CCT, mm	-0.001	0.924	0.010	0.524	-0.020	0.202	-0.005	0.760
AL, mm	-0.268	**0.000**	-0.282	**0.000**	-0.226	**0.000**	-0.222	**0.000**

RE, right eye; LE, left eye; LT, lens thickness; PD, pupil diameter; Kmean, mean corneal power; WTW, white-to-white distance; ACD, anterior chamber depth; CCT, central corneal thickness; AL, axial length

**Bold** indicates significant

**Fig. 6:**
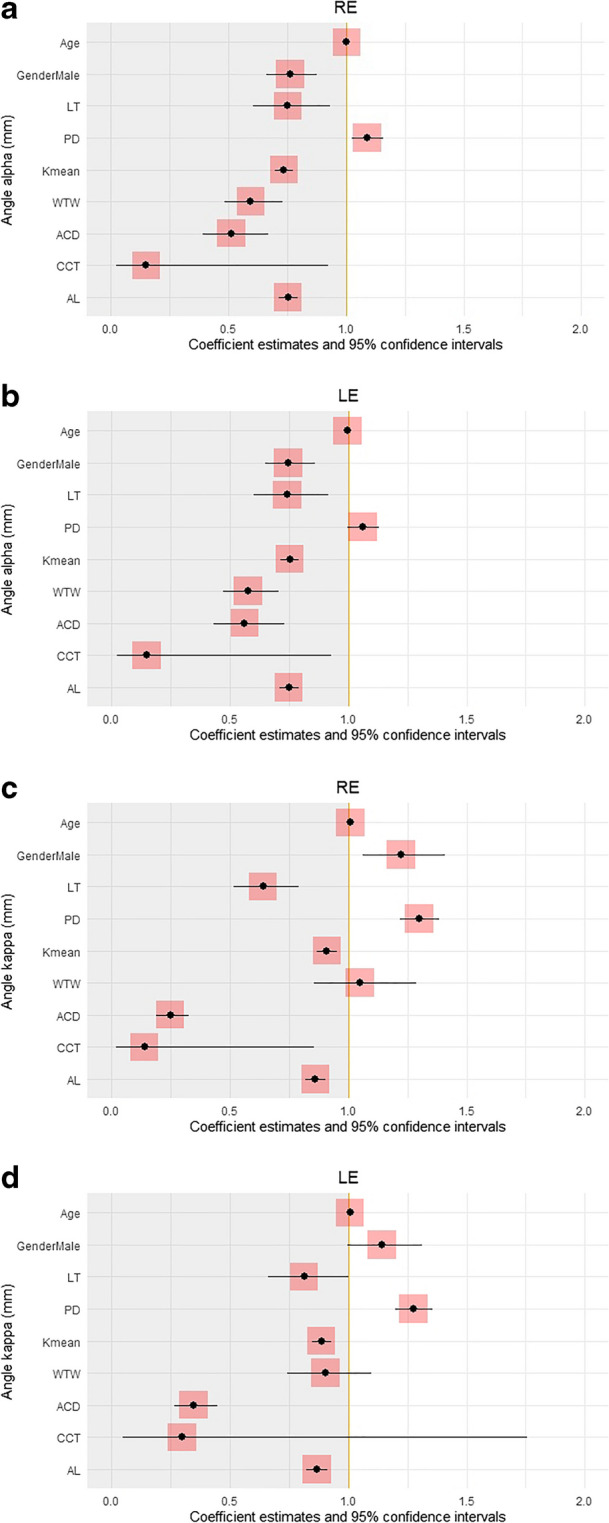
Multivariate logistic regression analysis offset magnitudes of (**a**) angle alpha in right eyes, (**b**) angle alpha in left eyes, (**c**) angle kappa in right eyes, and (**d**) angle kappa in left eyes. The odds ratio for each parameter to acquire an angle alpha of an offset magnitude >0.45 mm or an angle kappa of a offset magnitude >0.30 mm is shown, with corresponding confidence intervals. RE, right eye; LE, left eye; LT, lens thickness; PD, pupil diameter; Kmean, mean corneal power; WTW, white-to-white distance; ACD, anterior chamber depth; CCT, central corneal thickness; AL, axial length

A multivariate logistic regression analysis (Table 8 and Fig. 6) demonstrated that a greater offset magnitude of angle alpha was significantly associated with female gender, shorter LT, lower Kmean, smaller WTW, shallower ACD, thinner CCT, and shorter AL in both the right and left eyes individually (*P* < 0.05 for all). A greater offset magnitude of angle alpha was associated with a larger PD only in the right eyes, while a greater offset magnitude of angle kappa was associated with age, larger PD, lower Kmean, shallower ACD, and shorter AL for both eyes, and with male gender, shorter LT, and thinner CCT in right eyes only (*P* < 0.05 for all).

**Table 8:** Multivariate Logistic Regression Analysis of Parameters Associated with Angle Offset Magnitude

	Angle alpha offset, mm		Angle kappa offset, mm	
	RE	LE	RE	LE
Age, y	1.003 [0.997, 1.009], *P* = 0.349	0.998 [0.991, 1.004], *P* = 0.495	1.010 [1.004, 1.017], *P* = **0.002**	1.008 [1.002, 1.015], *P* = **0.012**
Male gender	0.764 [0.664, 0.878], *P* = **0.000**	0.747 [0.649, 0.860], *P* = **0.000**	1.223 [1.062, 1.408], *P* = **0.005**	1.143 [0.996, 1.311], *P* = 0.057
LT, mm	0.753 [0.608, 0.932], *P* = **0.009**	0.744 [0.602, 0.918], *P* = **0.006**	0.640 [0.517, 0.793], *P* = **0.000**	0.815 [0.664, 1.000], *P* = 0.050
PD, mm	1.090 [1.026, 1.159], *P* = **0.006**	1.063 [0.999, 1.131], *P* = 0.056	1.300 [1.220, 1.386], *P* = **0.000**	1.276 [1.200, 1.358], *P* = **0.000**
Kmean, D	0.736 [0.698, 0.776], *P* = **0.000**	0.754 [0.716, 0.794], *P* = **0.000**	0.910 [0.867, 0.954], *P* = **0.000**	0.887 [0.846, 0.930], *P* = **0.000**
WTW, mm	0.596 [0.485, 0.732], *P* = **0.000**	0.578 [0.472, 0.706], *P* = **0.000**	1.050 [0.855, 1.288], *P* = 0.642	0.904 [0.745, 1.099], *P* = 0.311
ACD, mm	0.514 [0.393, 0.671], *P* = **0.000**	0.563 [0.432, 0.732], *P* = **0.000**	0.249 [0.190, 0.326], *P* = **0.000**	0.347 [0.267, 0.450], *P* = **0.000**
CCT, mm	0.151 [0.025, 0.925], *P* = **0.041**	0.150 [0.024, 0.931], *P* = **0.043**	0.140 [0.023, 0.858], *P* = **0.034**	0.299 [0.050, 1.756], *P* = 0.183
AL, mm	0.755 [0.714, 0.796], *P* = **0.000**	0.750 [0.710, 0.792], *P* = **0.000**	0.861 [0.820, 0.904], *P* = **0.000**	0.869 [0.826, 0.912], *P* = **0.000**

RE, right eye; LE, left eye; LT, lens thickness; PD, pupil diameter; Kmean, mean corneal power; WTW, white-to-white distance; ACD, anterior chamber depth; CCT, central corneal thickness; AL, axial length

**Bold** indicates significant

## Discussion

There is paucity of literature on the potential role and measurements of angles alpha and kappa offset magnitude in cataract and refractive surgery, among populations worldwide. In this study, those values were measured with the IOLMaster 700 device in a large population of cataract surgery candidates.

In our study,there was no variation in angle alpha offset magnitude between the right and left eyes, in contrast to a significant variation in angle kappa offset magnitudes (Table 1 and Fig. 2). Since demographic and conventional ocular biometric parameters, including AL, Kmean, ACD, and LT, were similar between right and left eyes, this variation appears to represent a real difference and not a confounder effect. The variance in angle kappa offset magnitude could be further seen when stratified for gender and quadrants. Eye dominance, which was not measured in this study, may also be related to this finding, as demonstrated elsewhere [20]. Recently Mahr et al. collected angle alpha offset magnitudes of both right and left eyes but did not compare their offset magnitudes [21]. Instead, they found a positive correlation between right and left eyes.

AL-related differences in angle alpha were demonstrated and found to be higher in short eyes and very long eyes (Table 4). There were also gender-related differences in the offset magnitude of angle alpha, with an average larger one found in females (Table 6). Gender-related differences have been examined by others as well. Meng et al. [16] demonstrated gender-related difference in angle alpha offset magnitude in a Chinese population, but it is not clear whether angle alpha offset magnitude was greater in males or females due to ambiguity between their text and the related statistics. In contrast, Wang et al. [22] observed no significant gender-specific differences, a result that could be attributable to the relatively small sample size in their study (*n* = 81 eyes). Similar to angle alpha offset magnitude, AL-related differences in angle kappa offset magnitude were demonstrated and found to be higher in short eyes and very long eyes (Table 4). In contrast to angle alpha offset magnitude, no gender-related differences in the offset magnitude of angle kappa were demonstrated (Table 6), as reported by Meng et al. [16] and Wang et al. [22].

In the present study, the highest offset magnitudes of both angles were observed in short eyes, with a nonlinear trend of a gradual decrease in both angles’ offset magnitudes as AL increased, towards a nadir. In addition, there was a significant positive correlation of the offset magnitudes of angle alpha and angle kappa with each other. Similar findings were demonstrated by Basmak et al. [23] and by Meng et al. [16] Our findings make it clearer that the hyperopic patient is especially prone to high angle alpha and angle kappa offset magnitudes, which makes them especially challenging with laser-assisted in situ keratomileusis (LASIK), and probably with IOLs as well, even with EDOF type lenses.

The patient population of the present study was older than that of Meng et al. [16] (70.7 ± 12.4 vs 64.6 ± 11.9 years, respectively), and demonstrated larger right eye angle alpha (0.498 ± 0.198 vs 0.45 ± 0.21 mm, respectively) and angle kappa (0.368 ± 0.205 vs 0.30 ± 0.18 mm, respectively) offset magnitudes. Left eyes were not included in Meng et al.’s study [16]. Lopes et al. [24] demonstrated significantly greater angle kappa values in right eyes in an Italian population, non-significant differences in a Brazilian population, and significantly lower values in a Chinese population. It should be noted that those values cannot be compared to ours since Lopes et al. measured angle alpha and angle kappa by means of novel computational methods and reported these results in angular units. Langenbucher et al. [25] measured each cartesian coordinate of the offsets separately while grouping the right and left eyes together. Their preoperative measurements yielded an impressively smaller mean Ix value in comparison to our study (-0.006 vs. 0.436 mm, respectively) and a larger mean Iy value (0.131 vs. 0.046 mm, respectively). Px and Py values differences were similar. We assume that reasonable explanations of this significant variation are the mixture of both right and left eyes in the same group or a different method of calculation rather than using the crude analysis output of the IOLMaster 700. Using their own technique, they have tried to predict the post-operative CWC, using preoperative measurements, but reached unreliable results.

Kim et al. examined the repeatability of measurements taken by the IOLMaster 700, including chord mu, Px, Py, and pupil diameter, and compared them with those from Scheimpflug tomography (Pentacam HR, Oculus, Germany) [26]. The IOLMaster 700 exhibited superior repeatability, while the correlation between the two devices ranged from moderate to high. In another study, Zhang et al. found good agreement in angle kappa but poor agreement in angle alpha among three systems that used different technologies: Pentacam HR (Oculus, Germany), iTrace (Tracey Technology, Houston, Texas) and the IOLMaster 700 [27]. These findings are important to consider when using these parameters.

Cataract and refractive surgery with a monovision target approach require that one eye be allocated for each target distance. The conventional monovision approach is to correct the dominant eye for distance vision [28–30]. Kim et al. [20] demonstrated a smaller angle kappa in the dominant eye in a Korean population. In the present study, angle kappa offset magnitude was significantly greater in right eyes but, as noted earlier, we did not document eye dominance nor made a sub-group analysis of only patients with both eyes included. Further research aiming to find a relation between angle kappa’s offset magnitude and eye dominance is needed, with possible implication of its application as an objective marker to aid in choosing the eye more suitable for distance vs. near target, without the need of subjective optometric examination of eye dominance.

It is worth mentioning that Masket et al. [31], in their review of pseudophakic dysphotopsias, found an association between high positive angle kappa and high hyperopia, compatible with our findings. They noted that a high positive angle kappa and hyperopia are potentially in clinical association with negative dysphotopsias. Together with our findings, angle kappa values or offset magnitudes may be used as a cutoff when considering multifocal IOLs, as Mahr et al. [21] proposed using angle alpha alone. Indeed, the measurements norms are prone to be biometer-dependent, yet population-based baseline values can assist in eliminating the extreme values, or at least, require special attention and warning to the patients that they may be at higher risk due to pre-existing alignment issues, as part of their informed consent.

We demonstrated a strong association between angle alpha and angle kappa offset magnitudes, as well as variable associations between them and many biometric measurements. Since only several biometric devices currently report angle alpha and angle kappa offset magnitudes, reporting and analyzing these parameters and their correlations may reveal important future applications and possibly establish the necessity to assess both eyes before any ocular surgical intervention, in contrast to the current assumption of eye symmetry in these parameters. Moreover, recent studies have described a variety of relations between right and left eyes among various populations around the world [21, 24], which, in turn, can affect decision making regarding IOL-based refractive surgery as well as laser keratorefractive surgery.

The strengths of our study are our large population with a wide range of ages, and evidence to show that there is no symmetry between the right and left eyes. In addition, we measured angle alpha and angle kappa offset magnitudes of all patients with a single device, the IOLMaster 700 (a strength and a limitation simultaneously).

This study has several limitations, including its retrospective design, the inclusion of non-paired right and left eyes, and the absence of documentation regarding eye dominance. Ethnic differences that could not be addressed in this study due to its retrospective nature may warrant further exploration. Additionally, the angles are measured as offsets, and their clinical impact remains unclear. Angle lambda represents the deviation between the line of sight and the pupillary axis. Previous studies have suggested that a significant magnitude of angle lambda offset may lead to symptoms of dysphotopsia following trifocal IOL implantation [3]. However, the IOLMaster 700 does not currently assess this angle or its offset magnitude, which prevented its inclusion in our study.

In conclusion, the present study documents that the offset magnitudes of both the angle alpha and angle kappa present significant variations according to gender, eye laterality, angle location, and biometric parameters, such as AL. These values are also population-specific. Due to the importance of angle alpha and angle kappa for the satisfaction of patients after modern cataract and refractive surgery, understanding and subclassifying these angles and their offset magnitudes may improve patient selection and aid in choosing the most suitable personalized treatment options. Possible implications, such as refinement of objective identification of the dominant eye in monovision approach should be further inspected.
